# An Efficient Approach to the Accurate Prediction of Mutational Effects in Antigen Binding to the MHC1

**DOI:** 10.3390/molecules29040881

**Published:** 2024-02-16

**Authors:** Mengchen Zhou, Fanyu Zhao, Lan Yu, Jinfeng Liu, Jian Wang, John Z. H. Zhang

**Affiliations:** 1 Shanghai Engineering Research Center of Molecular Therapeutics and New Drug Development, Shanghai Key Laboratory of Green Chemistry & Chemical Process, School of Chemistry and Molecular Engineering, East China Normal University, Shanghai 200062, China; 51204300114@stu.ecnu.edu.cn; 2 NYU-ECNU Center for Computational Chemistry and Shanghai Frontiers Science Center of AI and DL, NYU Shanghai, 567 West Yangsi Road, Shanghai 200126, China; fz2113@nyu.edu; 3 Department of Basic Medicine and Clinical Pharmacy, China Pharmaceutical University, Nanjing 210009, China; 4 Faculty of Synthetic Biology and Institute of Synthetic Biology, Shenzhen Institute of Advanced Technology, Chinese Academy of Sciences, Shenzhen 518055, China; jian_wang@post.harvard.edu; 5 Department of Chemistry, New York University, New York, NY 10003, USA; 6 Collaborative Innovation Center of Extreme Optics, Shanxi University, Taiyuan 030006, China

**Keywords:** peptide, MHC, binding free energy, alanine scanning, point mutations, hotspot residues

## Abstract

The major histocompatibility complex (MHC) can recognize and bind to external peptides to generate effective immune responses by presenting the peptides to T cells. Therefore, understanding the binding modes of peptide–MHC complexes (pMHC) and predicting the binding affinity of pMHCs play a crucial role in the rational design of peptide vaccines. In this study, we employed molecular dynamics (MD) simulations and free energy calculations with an Alanine Scanning with Generalized Born and Interaction Entropy (ASGBIE) method to investigate the protein–peptide interaction between HLA-A*02:01 and the G9_209_ peptide derived from the melanoma antigen gp100. The energy contribution of individual residue was calculated using alanine scanning, and hotspots on both the MHC and the peptides were identified. Our study shows that the pMHC binding is dominated by the van der Waals interactions. Furthermore, we optimized the ASGBIE method, achieving a Pearson correlation coefficient of 0.91 between predicted and experimental binding affinity for mutated antigens. This represents a significant improvement over the conventional MM/GBSA method, which yields a Pearson correlation coefficient of 0.22. The computational protocol developed in this study can be applied to the computational screening of antigens for the MHC1 as well as other protein–peptide binding systems.

## 1. Introduction

The major histocompatibility complex (MHC) can present pathogen-derived peptides or disease-associated protein fragments to T cells, which is a prerequisite for T cells to initiate specific immune responses against pathogens or abnormal cells [1]. The MHC system is divided into two primary classes: Class I and Class II. Class I MHC molecules (MHC1), recognized by CD8^+^ T cells, are more widely distributed and are expressed on the surface of all nucleated cells, generally presenting endogenous antigens produced by normal or abnormal cellular metabolism. In contrast, Class II MHC molecules (MHC2), identified by CD4^+^ T cells, are expressed only in antigen-presenting cells, primarily recognizing exogenous antigens, such as bacteria or other pathogens [2]. In this study, we focused on the MHC1, a heterodimer consisting of an α-chain and a β_2_-microglobulin (Figure S1a). The MHC1 binds to short peptides (mainly 8–10 amino acids) with a binding groove enclosed by two α-helices and seven antiparallel β-strands (Figure S1b). There are some key binding sites for peptides bound to the MHC1 where the residues on the peptides involved are called anchor residues. These anchor residues are predominantly close to the N-terminus and the C-terminus of the peptides (Figure S1c), whereas the residues arching in the middle are more relevant for T cell receptors (TCR) interactions [3,4].

Endogenous antigens are processed intracellularly into peptide fragments that bind to the MHC1, and the complex is further presented to the cell membrane for recognition by T cells [1]. According to this mechanism, we can design peptide vaccines to activate T cells, thereby inducing specific immune responses to prevent or treat a wide range of diseases, including infections and tumors. Whether MHC molecules can present antigens depends on the affinity between them, but many epitopes generated from self-proteins exhibit a low binding affinity with the MHC. Hence, we can mutate anchor residues near the N- and C-termini to enhance the affinity of the peptide–MHC (pMHC) complex [5,6].

Currently, several computational methods have been developed to quantitatively predict the binding energies of mutant peptides and the MHC, which can decrease the time and computational costs significantly compared to experimental methods. There are two main categories of computational methods: data-based prediction methods and structure-based methods. Data-based prediction methods are divided into three main categories [7]: sequence scoring function-based algorithms [8,9], machine learning-based algorithms [10,11], and integration algorithms of different predictors [12,13]. The data-based prediction methods are rapid and well suited to high-throughput screening. However, they demand a large volume of accurate training data. Their predictive performance is notably higher for data like the training set, but their extrapolation capabilities may be limited. On the other hand, methods based on molecular dynamics (MD) simulations exhibit enhanced extrapolation performance. MD methods can be broadly classified into two groups. The first group includes alchemical methods grounded in a rigorous theoretical framework. Commonly used alchemical methods include the thermodynamic integration (TI) [14], the free energy perturbation (FEP) [15], the Bennett acceptance ratio (BAR) [16], and the multistate Bennett acceptance ratio (MBAR) [17]. These techniques are highly accurate and have been increasingly utilized. Nevertheless, their computational costs are high, and the results become less accurate when a net charge change occurs between the two states involved [18,19]. The second group primarily utilizes implicit solvation models such as the molecular mechanics generalized Born surface area (MM/GBSA) and the molecular mechanics Poisson−Boltzmann surface area (MM/PBSA) [20,21]. These methods are favored for their computational efficiency, though their accuracy is often contingent on the specific systems being studied.

Building upon the MM/GBSA method, the Alanine Scanning with Generalized Born and Interaction Entropy (ASGBIE) method is proposed for computing binding free energies [22,23]. In this approach, the Alanine Scanning method (AS) is used to calculate the contribution of residues at the interface to the binding energy, and the enthalpy and entropy changes of individual residues are calculated using the MM/GBSA (GB) and Interaction Entropy (IE) [24], respectively. An advantage of this approach lies in its ability to identify key residues [25,26] (hotspots) at the binding interface that contribute significantly to binding. Analysis of these hotspot residues provides deeper insights into the binding mode of the system, enhancing our understanding of its mechanism of action. Furthermore, ASGBIE facilitates the comprehensive analysis of the energy contributions, elucidating, for instance, whether the dominant contributions arise from electrostatic energies or van der Waals energies. The information obtained from this analysis can inform the rational design of mutations toward affinity-enhanced proteins or peptides.

In this study, we employed the ASGBIE method to investigate the binding mode of the pMHC1. We selected a representative pMHC1 system in which the MHC and the peptide were HLA-A*0201 and G9_209_, respectively. G9_209_ is a fragment of the melanoma antigen gp100, which has been used in the development of cancer vaccines and has significant value. Our study will first analyze and identify hotspots on the peptide antigen and the MHC1 by MD simulation and by using the ASGBIE method. Through energy decomposition, we will investigate the nature of the interaction in antigen binding to the MHC1. Such analysis will help us modify the computational method to further improve the accuracy of calculations. An optimized ASGBIE method is proposed to provide a more accurate prediction of peptide mutations in peptide–MHC1 systems.

## 2. Results and Discussion

**Figure 1:**
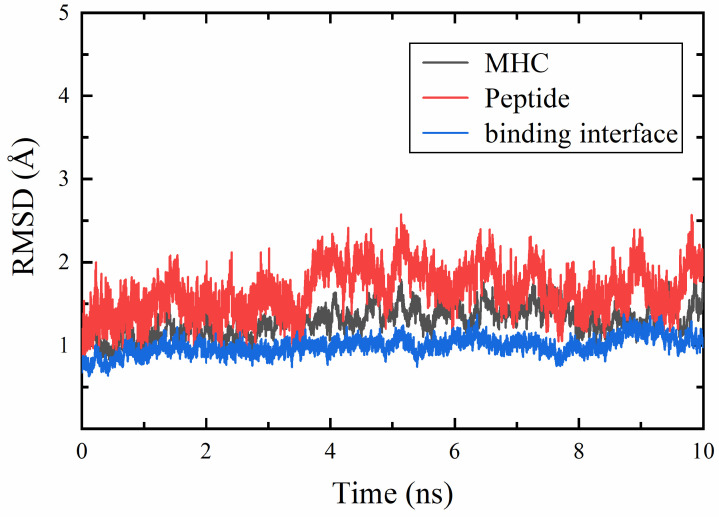
RMSD of the MHC, peptide, and the binding interface in the wild-type system.

We separately calculated the root mean square deviations (RMSD) of the MHC, the peptide, and the binding interface (the peptide and the MHC binding groove) relative to the initial structures (Figure 1) to assess the stability of the simulations. Both the MHC and the peptide exhibited fluctuations of less than nearly 2 Å, indicating the simulations remained stable. Furthermore, in comparison to the entire system, the RMSD of the binding interface significantly decreased, suggesting the stability of the binding.

### 2.1. Analysis of Hotspot Residues on Peptide

**Figure 2:**
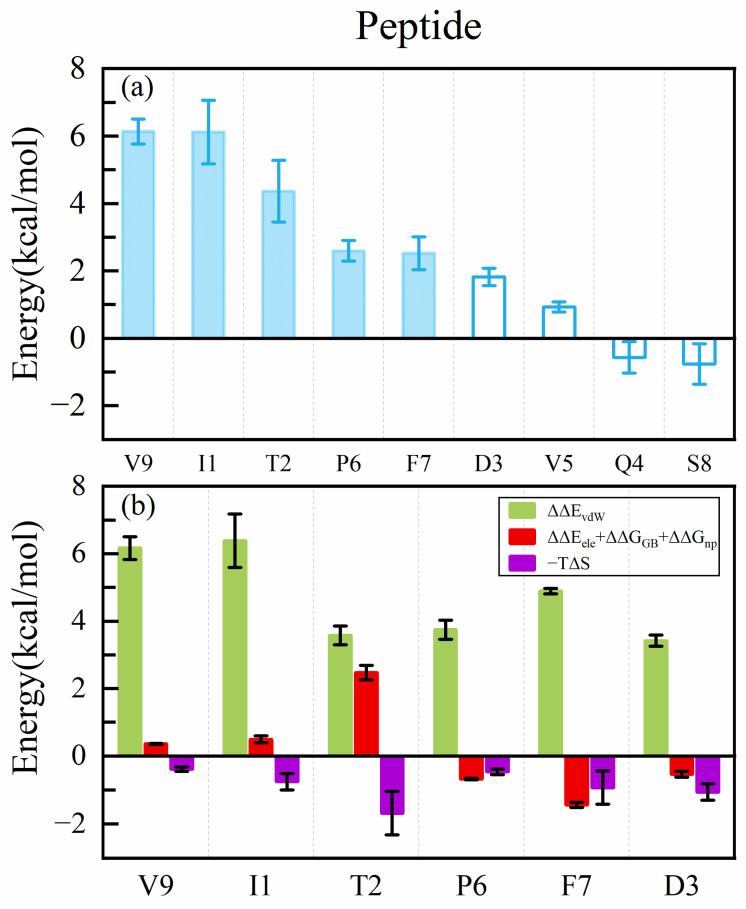
(**a**) Binding free energy contribution of residues on the peptide. (**b**) Decomposition of binding free energy contribution of hotspot residues on the peptide.

**Figure 3:**
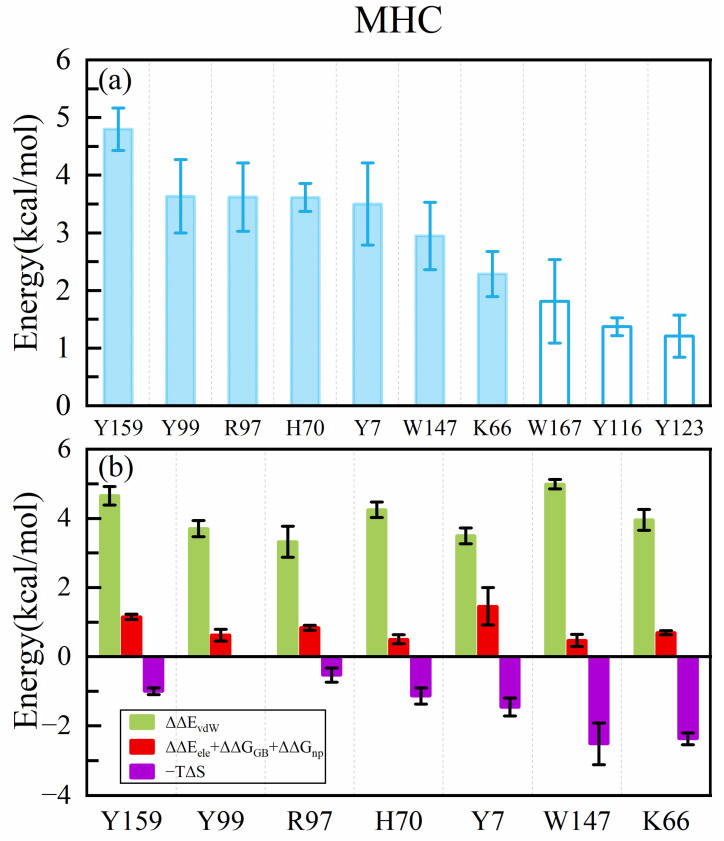
(**a**) Binding free energy contribution of residues on the MHC. (**b**) Decomposition of binding free energy contribution of hotspot residues on the MHC.

The binding of the MHC to the peptide is a crucial step in initiating the immune response. Therefore, studying the interaction between antigens and the MHC has significant implications for peptide design. To investigate the binding mode of the wild-type peptide ITDQVPFSV with HLA-A*02:01, we utilized the ASGBIE method to identify hotspots on the peptide and the MHC (Figure 2a and Figure 3a).

We first observed the hotspot residues on the peptide, and for clearer comparison, residues on the peptide were numbered from one to nine (corresponding to residues 376–384). Figure 2a illustrates that the contributions to binding free energy exceed 2 kcal/mol for the residues I1, T2, P6, F7, and V9, primarily located at both ends of the peptide. Notably, the C-terminal residue V9 and the N-terminal residue I1 exerts the highest energy contribution, measuring 6.13 ± 0.37 kcal/mol and 6.12 ± 0.94 kcal/mol, respectively. The findings in Figure 2b reveal that van der Waals interactions are favorable for binding energy and are the predominant contributor to the binding energy of all hotspot residues. In contrast, entropy changes adversely impact all hotspot residues.

**Figure 4:**
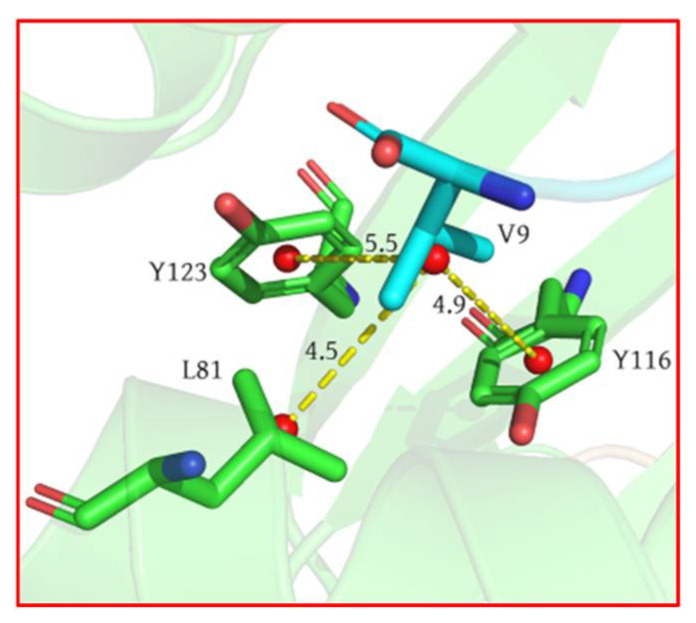
V9 on the peptide and L81 on MHC exhibit CH–CH interactions, while Y116 and Y123 engage in CH–π interactions. Residues are color coded according to their source, with green representing MHC and blue representing the peptide.

To investigate the sources of van der Waals energy from each hotspot residue more comprehensively, we performed van der Waals interaction calculations for atoms between the peptide and the MHC groove (see Table S4). Combining crystal structures, we elucidated the significant interactions of the hotspot residues. As depicted in Figure 4, for the C-terminus of the peptide, the alkyl group on residue V9 engages in CH–π interactions with the hydrophobic residues Y116 and Y123 and CH–CH interactions with L81. Meanwhile, at the N-terminus of the peptide, the alkyl group on residue I1 forms a CH–π interaction with hydrophobic residue W167 and a simultaneous CH–CH interaction with T163. These interactions anchor both ends of the peptide within the MHC groove. The third-highest contributing residue to the binding energy is T2. The hydroxyl group on the side chain of T2 forms a highly stable hydrogen bond with E63, which is the only stable hydrogen bond formed on a peptide side chain. This explains why T2 is the sole hotspot residue whose electrostatic interactions contribute positively to the binding energy.

By analyzing the hotspot residues on the peptide, our results align with the prior experience that the MHC is specifically recognized mainly at both ends of the peptide. We also found that van der Waals interactions play a predominant role in binding.

### 2.2. Analysis of Hotspot Residues on MHC1

Following this, we conducted an alanine scanning for the residues on the MHC binding interface (within 5 Å of the peptide). Y159, Y99, R97, H70, Y7, W147, and K66 (Figure 3a) were found to contribute more than 2 kcal/mol to the binding energy. Notably, these residues, except for R97 and K66, comprise amino acids with aromatic rings. Additionally, it was observed that most residues in the binding interface possessed hydrophobic side chains. Energy decomposition results (Figure 3b) indicated that van der Waals interactions predominantly contributed to the binding energy, which was consistent with the peptide results.

**Figure 5:**
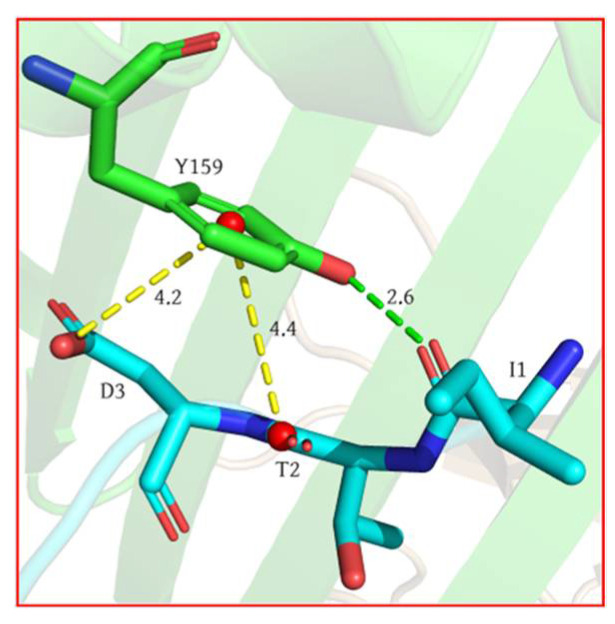
Y159 on the MHC and D3 on peptide exhibit Anion–π interaction, along with amide–π stacking interactions with T2 and D3, and a hydrogen bond with I1. Residues are color coded according to their source, with green representing MHC and blue representing the peptide.

It was revealed that, in addition to the interactions between peptide side chains and the MHC, numerous interactions occurred between the MHC and the peptide’s main chain. Specifically, the side chain of Y159 could form an Amide–π stacked interaction with the amide bonds between T2 and D3, as well as hydrogen bonds with the carbonyl oxygen atoms on I1’s main chain (Figure 5). Similarly, H70 could engage in Amide–π stacked interactions between Q4 and V5, along with forming hydrogen bonds with the carbonyl oxygen on D3. Results from the van der Waals energy calculations suggested that side chain van der Waals interactions accounted for roughly half of all van der Waals interactions (see Figure S2).

These findings also provide insights into the calculation of the binding energy between the peptide and the MHC. Van der Waals interactions play a dominant role in this system, suggesting their potential utility in evaluating the system’s binding energy. Furthermore, it may be necessary to simultaneously perform alanine scanning both on the MHC and the peptide to obtain the final binding energy, allowing for a comprehensive assessment of interactions between all atoms in the complex.

### 2.3. Free Energy Calculation Using ASGBIE Method

To quantitatively predict the impact of peptide mutations on the pMHC1 binding affinity, we conducted six independent 10 ns simulations for all complexes. From each set of simulations, we used the last 9 ns trajectory for binding free energy calculations. When we use alanine scanning for binding energy calculations, as discussed in Section 2.1 and Section 2.2, we have the option to perform alanine scanning either on the MHC (MHC–AS) or on the peptide (Peptide–AS). In this study, the experimental binding affinities were derived using the ΔG_exp_ = RTlnID_50_ formula, providing a basis for comparison with our computationally determined binding free energies. Then we computed the correlation between the averaged results from the six sets and the experimental binding affinity. Table 1 presents the Pearson correlation coefficients (*r*_P_), while the Spearman ranking correlation coefficients (*r*_S_) are demonstrated in Table S5. All of the averaged calculation energies in this section including the individual energy components were directly performed correlation calculations with the experimental binding affinity.

Upon breaking down the binding free energy into distinct energy components, we found that the correlation of the van der Waals term alone (Δ*E*_vdW_) reached as high as 0.77, which is higher than the value of ∆*H*, the sum of the van der Waals energy, the electrostatic energy (∆*E*_ele_), and the solvation energies (∆*G*_sol_). This observation aligns with the premise that van der Waals interactions are the main component of the free energy differences, as demonstrated in previous studies by Wang et al. [27], where this term alone achieved a notably high correlation. This is also consistent with the results of our previous analysis of the wild-type system. Table 1 shows the correlation between the Δ*E*_vdW_-*T*Δ*S* results improving from 0.77 to 0.86 compared to Δ*E*_vdW_, and Δ*G* improving compared to Δ*H*. This agrees with many previous results in the literature that adding the entropy term calculated by the IE leads to a better agreement with the experimental data and can be closer to the absolute free energy [23,28].

Our results revealed that when the MHC–AS and the Peptide–AS were used separately, the correlations of the ∆*E*_vdW_-*T*∆*S* term were 0.77 and 0.86, respectively, with the Peptide–AS method demonstrating superior performance. This can be attributed to the fact that mutations occur on the peptide, so alanine scanning for peptides can reflect the mutation effect more precisely. In addition, since the MHC–AS method involves the calculation of more residues, the standard deviation of the MHC–AS results between trajectories was relatively large (Table S1).

**Figure 6:**
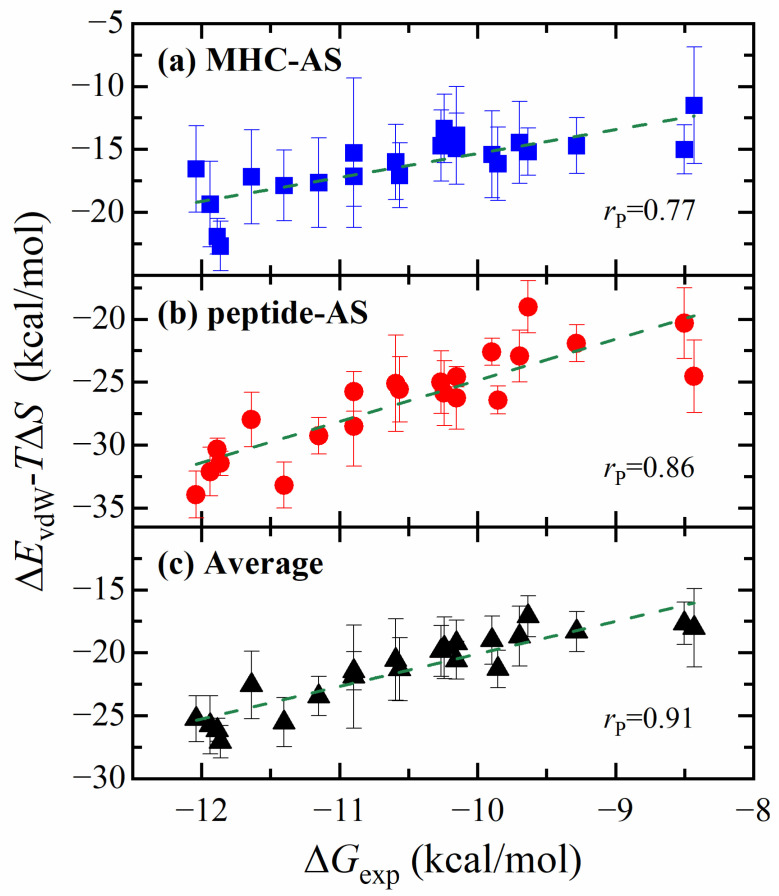
Correlations between experimental binding affinity and computed energies from different alanine scanning methods: (**a**) MHC–AS, (**b**) Peptide–AS, (**c**) Average method. Three different colors represent the calculated energies for the three methods, and the green dashed line represents the linear fit curve. The averaged Δ*E*_vdW_-*T*Δ*S* energies of all trajectories are compared with the experimental binding energies Δ*G*_exp_.

In addition to the MHC–AS and Peptide–AS methods, we can also average the two sets of results obtained, as mentioned in the Methods section. Figure 6 displays the correlation between the calculated values from the three methods and the experimental values. It is evident that the Average method demonstrates superior performance, with the correlation coefficient increasing to 0.91. This improvement was also verified through multiple rounds of bootstrap resampling (Figure S3). The observed improvement can be attributed to the complementary effects of MHC–AS and Peptide–AS. Figure 6 presents the scatter plot of the three computational methods, indicating that some predicted values were underestimated in the MHC–AS method and overestimated in Peptide–AS, or vice versa, such as the right two points in the plot.

This complementarity may come from the fact that the results of Peptide–AS can only calculate interactions on the side chains of the peptide, thereby excluding the CB (beta carbon) and attached hydrogens. When using alanine scanning to calculate the residues on the peptide, we turn the side chains of the residues into methyl in the trajectory. This substitution only captures the contributions of the peptide side chains to the binding energy, which neglects the contributions of the peptide’s CB and main chain. However, the result of MHC–AS is to turn the side chain of the residue on the MHC into methyl, which completely calculates the binding energy of the side chain of the MHC to all atoms on the peptide. Therefore, when we average the MHC–AS results with the Peptide–AS results, the contributions of all atoms on the peptide are included in the calculation. This may explain why the average result is better than both individual results.

In summary, the Average method harnessed the advantages of both the MHC–AS and the Peptide–AS methods, leading to the achievement of a more comprehensive calculation with improved accuracy and reduced standard deviation. Thus far, we have found two paths for optimizing the ASGBIE method for the pMHC system: calculating only the van der Waals energy change and entropy change and averaging the results of alanine scanning from both MHC–AS and Peptide–AS. Next, we compared our optimized ASGBIE method with the MM/GBSA method, the original ASGBIE method, and the more precise alchemical method.

**Figure 7:**
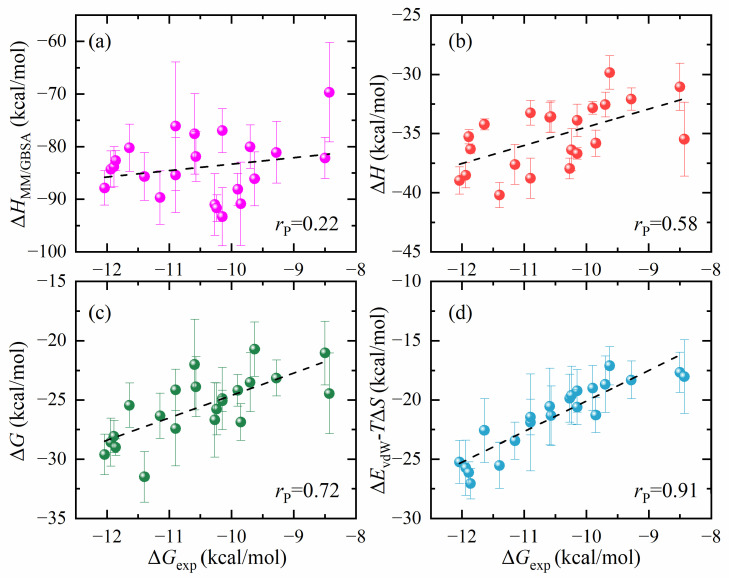
Correlation between experimental binding affinity and computed energies from different energy calculation methods: (**a**) Δ*H*_MM/GBSA_ calculated by MM/GBSA method, (**b**) Δ*H* calculated by the ASGBIE method, (**c**) Δ*G* calculated by the ASGBIE method, and (**d**) Δ*E*_vdW_-*T*Δ*S* calculated by the optimized ASGBIE method. The different colors represent the calculated energies for the four methods, and the black dashed line represents the linear fit curve.

The computational outcomes obtained using the MM/GBSA method, the ASGBIE method, and the optimized method are presented in Figure 7. The correlation coefficient calculated with the MM/GBSA method (Δ*H*_MM/GBSA_) is relatively low (*r*_P_ = 0.22 and *r*_S_ = 0.12), whereas the correlation coefficient of the enthalpy change (Δ*H*) calculated using the ASGBIE method is substantially higher (*r*_P_ = 0.58 and *r*_S_ = 0.62), and the standard deviation was also significantly reduced. When the entropy change results are incorporated, the correlation coefficients for ΔG calculated via the ASGBIE method show marked improvement (*r*_P_ = 0.72 and *r*_S_ = 0.73). However, the correlation of our optimized ASGBIE method is further improved (*r*_P_ = 0.91 and *r*_S_ = 0.94), which is much higher than the results of the MM/GBSA and the original ASGBIE.

### 2.4. Comparison with Alchemical Method

**Figure 8:**
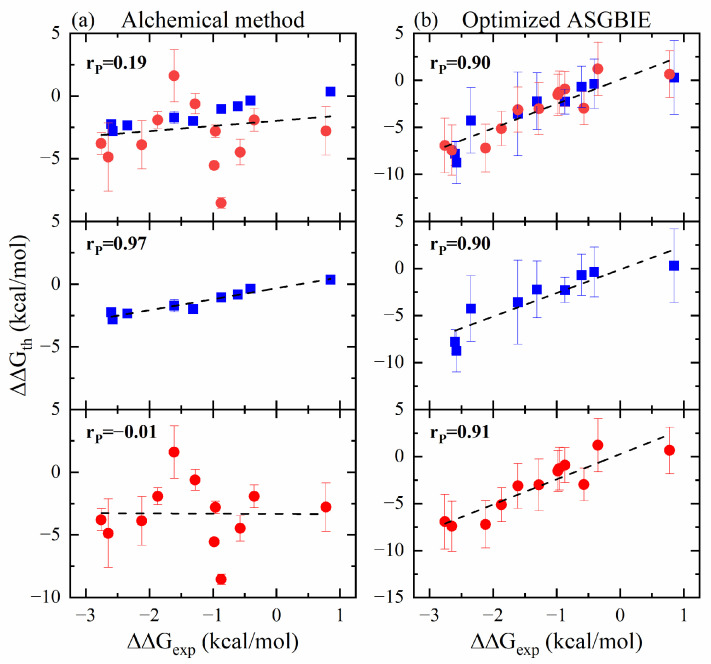
Scatter plots of the theoretical calculated values from (**a**) the alchemical method, (**b**) the optimized ASGBIE method. The scatter plots are colored according to the presence or absence of charge-changing mutations, with unchanged mutations in blue squares and changed mutations in red dots, and the black dashed line represents the linear fit curve. The results of the alchemical method use the average of the four methods (FEP, TI, BAR, MBAR), and the error values are the standard deviations of these methods. Since the alchemical method yields relative free energies, for comparison the computed values in the optimized ASGBIE methods and experiment values here all use the relative energy.

We used four alchemical protocols to predict the relative binding free energy upon mutations [29]. Figure 8 shows the relative binding energies obtained from the alchemical method and the optimized ASGBIE method as well as the Pearson correlation with the experimental measurements. The Spearman correlation is demonstrated in Tables S6 and S7. The overall Pearson correlation of 0.9 for the optimized ASGBIE method is much higher than that of 0.19 for the alchemical methods. Among the 21 mutations we tested, net charge changing was involved in 12 pairs of mutations, while it was not observed in the other 9 mutations. The blue squares in Figure 8 are the binding affinity of the 9 variants without net charge changing. The Pearson correlation between the experimental and calculated values of the alchemical method was extremely high, at 0.97. It demonstrates that all the alchemical protocols perform excellently without net charge changing involved.

The red dots in Figure 8 are the predicted relative binding energies for the 12 mutations with net charge variations. The red dots corresponding to the results from the alchemical methods are widely scattered, significantly reducing the overall correlation. This observation indicates that the alchemical methods cannot accurately predict the relative binding free energy of charged mutations. This inaccuracy can be attributed to several things that we detail and suggest possible solutions to in the Supporting Information. On the other hand, the net charge changing had almost no effect on our proposed scheme, and the correlation remained above 0.9. Hence, compared to the alchemical approach, the optimized ASGBIE method is capable of a more comprehensive prediction of the affinity of the pMHC upon point mutations.

## 3. Methods

### 3.1. MD Simulations

To better understand the binding mechanism between the MHC and the peptide and to explore optimal strategies for calculating mutational binding energy, we selected a system that incorporates 12 single-point and 9 double-point mutational (spread over N-terminal anchor residues, see Tables S1–S3) experimental data [30]. The wild-type system is HLA-A*02:01-G9_209_ (PDB id: 1TVB) and the structure was downloaded from the RCSB PDB website (http://www.rcsb.org) on 1 October 2022. We used the TLEaP from AmberTools v18 [31] to mutate the peptides bound in the MHC groove. All charged residues in the systems were assigned their charge states corresponding to physiological pH (7.4).

All systems were performed under an AMBER ff14SB force field [32]. Each pMHC complex was solvated in a periodic box containing TIP3P [33] water molecules with a minimum buffering distance of 15 Å between any atom of the complex and the box surfaces. Counter ions were added to neutralize the systems. The Langevin dynamics [34] was used to regulate the temperature with a collision frequency of 1.0 ps^−1^, while the pressure was controlled to 1.0 atm by the Berendsen barostat [35]. The particle mesh Ewald (PME) [36] algorithm was used to treat long-range electrostatic interactions, and the nonbonded cutoff was set to 10 Å. The SHAKE [37] algorithm was used to constrain bonds involving hydrogen atoms. Each system was minimized with steepest descent cycles followed by conjugate gradient cycles. During minimization, the coordinates of protein atoms were first restrained by a force constant of 500 kcal/(mol·Å^2^) to allow the solvent molecules to adequately cater to the complex. The constraints were then removed to optimize the whole system and especially to eliminate collisions that may be caused by mutations. The systems were then slowly heated to 300 K within 300 ps in an NVT ensemble, and all protein atoms were constrained. Then a 1 ns equilibration was conducted using the NPT ensemble and with restraints on protein atoms. Finally, 10 ns production was run without any restraints, and the trajectory was saved for every picosecond. The last 9 ns trajectory was used to calculate the binding free energy. All 9000 frames obtained were used for entropy change calculations, and 100 snapshots were equally spaced and extracted for enthalpy change calculations.

### 3.2. ASGBIE Method

#### 3.2.1. Alanine Scanning

In our alanine scanning approach, the contribution of the mutated residues (*x*) to the binding energy is given by the difference in binding free energy before and after the mutation, which can be expressed as

(1) $$\begin{array}{c} \Delta \Delta G_{\mathrm{bind}}^{x\to a}=\Delta G_{\mathrm{bind}}^{a}-\Delta G_{\mathrm{bind}}^{x} \end{array}$$

where $\Delta G_{\mathrm{bind}}^{a}$ and $\Delta G_{\mathrm{bind}}^{x}$ are the binding free energies of the alanine-mutant and the wild-type complex, respectively. Equation (1) can be broken down into different components,

(2) $$\begin{array}{c} \Delta \Delta G_{\mathrm{bind}}^{x\to a}=\Delta \Delta E_{\mathrm{internal}}^{x\to a}+\Delta \Delta E_{\mathrm{vdW}}^{x\to a}+\Delta \Delta E_{\mathrm{ele}}^{x\to a}+\Delta \Delta G_{\mathrm{sol}}^{x\to a}-T\Delta \Delta S^{x\to a} \end{array}$$

where ∆*E*_internal_, ∆*E*_vdW_, ∆*E*_ele_, ∆*G*_sol_, and *T*∆*S* represent, respectively, the internal energy, the van der Waals interaction, electrostatic interaction, solvation free energy, and the entropy contributions [38]. As we used the 1-trajectory method, the changes in internal energy can be effectively canceled out. The dielectric constants of nonpolar, polar, and charged specific residues were set to 1, 3, and 10 respectively [39].

Although a pMHC interface typically contains tens of residues, only a few important residues contribute to binding in the interface [25,26]. We assumed that if residues outside 5 Å of the binding interface contribute weakly to the interaction, then the total binding energy can be obtained by summing up the contributions of individual residues on the MHC or the peptide within 5 Å:

(3) $$\Delta G_{\mathrm{bind}}=-\underset{x}{\sum }\Delta \Delta G_{\mathrm{bind}}^{x\to a}$$

where the summation is overall contributing residues *x* in one of the binding partners.

**Figure 9:**
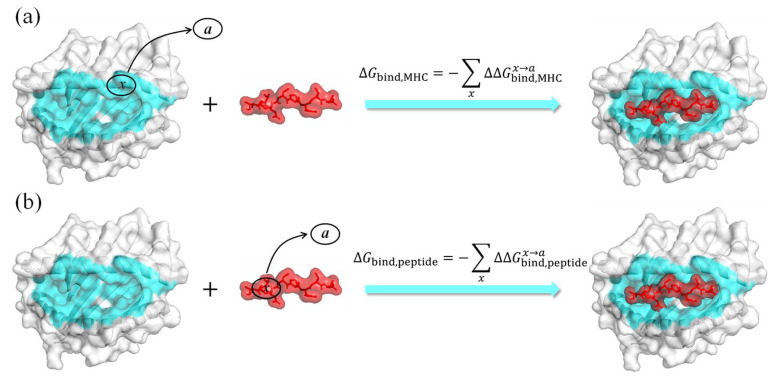
Different approaches to the alanine scanning process, (**a**) mutating residues on MHC within 5 Å, (**b**) mutating residues on peptide.

As illustrated in Figure 9, two binding free energy values can be obtained by alanine scanning of residues on the MHC (MHC–AS) or on the peptide (Peptide–AS).

#### 3.2.2. IE Method for Entropy Calculation

The calculation of the change in entropy (−*T*Δ*S*) is usually performed by normal-mode analysis. However, because of the heavy computational cost, most GB or PB calculations neglect the entropy calculation. Here, we used the IE method to calculate the entropy contribution in the alanine scanning method [23]. The formula for IE can be derived as follow:

(4) $$\begin{array}{c} -T\Delta S=KTln\langle e^{\beta \Delta E_{\mathrm{int}}}\rangle \end{array}$$

where *β* denotes 1/*KT* and *E*_int_ is the interaction energy between the MHC–peptide, which includes both *E*_ele_ and *E*_vdW_ interactions, and ∆*E*_int_ represents the fluctuation of the interaction energy. <$e^{\beta \Delta E_{\mathrm{int}}}$> means ensemble average, which can be readily obtained from MD trajectories without incurring additional computational expenses. And we applied an energy cutoff limit of 3σ to eliminate the energy “noises” in the IE calculation [23,40].

#### 3.2.3. Optimized ASGBIE Method

Although Equation (3) can be used to calculate binding energies using free energy contributions from hotspot residues either on the peptide or the MHC1, the numerical results are not the same. Here, we propose an optimized ASGBIE method by taking the average of both calculations:

(5) $$\Delta G_{\mathrm{bind}}=-\frac{1}{2}\left(\underset{x}{\sum }\Delta \Delta G_{\mathrm{bind},\mathrm{MHC}}^{x\to a}+\underset{x}{\sum }\Delta \Delta G_{\mathrm{bind},\mathrm{peptide}}^{x\to a}\right)$$

Also, if the peptide–MHC1 interaction is dominated by hydrophobic interactions, which is indeed the case as shown in the Results section, neglecting the electrostatic interaction energy can provide a more accurate result. This is executed by neglecting the third (electrostatic) and fourth (solvation) energy terms in Equation (2) in the summation of Equation (3):

(6) $$\begin{array}{c} \Delta \Delta G_{\mathrm{bind}}^{x\to a}\approx \Delta \Delta E_{\mathrm{vdW}}^{x\to a}-T\Delta \Delta S^{x\to a} \end{array}$$

The reason for neglecting electrostatic and solvation energies in protein–peptide systems dominated by hydrophobic interactions is the large inaccuracies in current electrostatic energy calculations. These errors may stem from the point charge model, the incorporation of fixed charges, and the use of implicit solvent models.

## 4. Conclusions

We carried out a computational study of antigen (peptide) binding to the MHC1 (HLA-A*02:01-G9_209_ system), identified hotspots on both the antigen and the MHC1, and accurately predicated the effect of peptide mutations on antigen–MHC1 binding free energy. The main results of the current study are summarized below.

1. Our calculation showed that the two terminal residues of the peptide contributed the most to the peptide binding to the MHC1, with interactions dominated by the van der Waals interaction, followed by the no. 2 (threonine) residue. The antigen–MHC1 binding is dominated by hydrophobic interactions.
2. The quantitative effect of the peptide (antigen) mutation on the antigen–MHC1 binding free energy is accurately predicted by the ASGBIE method with a good correlation to the experimental data. In particular, when electrostatic interactions (together with solvation) are neglected, the accuracy of the ASGBIE calculation is further improved. This finding suggests that for antigen–MHC1 bindings primarily governed by hydrophobic interactions, inaccuracies inherent in the point charge representation of electrostatic interactions within the force field and those in the implicit solvation model can detrimentally affect the accuracy of the results.
3. The ASGBIE method is optimized by using an averaged version, i.e., averaging the summation over residues on both interacting partners. The accuracy of this optimized ASGBIE result is further improved. This enhancement stems from the complementary nature of the MHC–AS and Peptide–AS methods.
4. The antigen–MHC1 binding energy calculation using the standard MM/GBSA method does not result in a good correlation with the experimental result. We also performed alchemical calculations of binding energy for comparison and the results are mixed. For peptide mutations that do not involve changes in electric charge, the alchemical calculation results in a very good correlation with the experimental result. However, for charge-changing mutations, the alchemical calculations result in significant errors. This discrepancy is a recognized limitation in alchemical methods involving a change of electric charge between the initial and the final states.

## Figures and Tables

**Table 1:** Pearson correlation coefficient for each computed energy with experimental binding affinity under different alanine scanning methods. MHC–AS means the ASGBIE calculation is calculated based on the summation over MHC residues, and similar definition holds for Peptide–AS method.

Energy Component	Pearson Correlation Coefficient	
	MHC-AS	Peptide-AS
Δ*H*_MM/GBSA_	0.22	0.22
Δ*E*_vdW_	0.73	0.77
Δ*H*	0.30	0.58
Δ*G*	0.46	0.72
Δ*E*_vdW_-*T*Δ*S*	0.77	0.86

## Data Availability

All MD files used in this study were deposited at https://github.com/kail0221/mutational_effect accessed on 6 October 2023. This deposition includes the following: (1) structure preparation files including tleap scripts, initial structure files, and initial parameter files, (2) input files used for molecular dynamics simulations, and (3) analysis and statistical data scripts.

## Supplementary Materials

The following supporting information can be downloaded at: https://www.mdpi.com/article/10.3390/molecules29040881/s1, 1. Effect of the number of trajectories on convergence; 2. Reasons for the inaccuracy of the alchemical method; Figure S1: (a) Overview of the peptide-MHC1 complex (PDB id: 1TVB). (b) Structure of the binding interface (includes peptide and MHC1 binding groove). (c) Structure of the binding site and the anchor residue; Figure S2: The vdW interactions between peptide hotspot residues and MHC groove residues: blue represents contributions from peptide side chain atoms (excluding CB and attached hydrogens), red represents contributions from all atoms in peptide residues; Figure S3: Bootstrapping to access the effect of the number of replicated trajectories on the Pearson correlation coefficient and its standard deviation; Table S1: Experimental binding free energy and calculated data for MHC-AS method with wild-type and mutants; Table S2: Experimental binding free energy and calculated data for peptide-AS method with wild-type and mutants; Table S3: Experimental binding free energy and calculated data for Average method with wild-type and mutants; Table S4: The vdW interaction of peptide side chain with MHC groove; Table S5: Spearman correlation coefficient for each energy with experimental binding affinity under different alanine scanning methods. MHC-AS means the ASGBIE calculation is calculated based on the summation over MHC residues, and similar definition holds for Peptide-AS method; Table S6: The experimental and calculated data of relative binding free energy of 9 mutations without net charge-changing; Table S7: The experimental and calculated data of relative binding free energy of 12 mutations with net charge-changing [41,42,43,44,45].
