# Supplementary material for: An Efficient Approach to the Accurate Prediction of Mutational Effects in Antigen Binding to the MHC1
Source: Molecules. 2024 Feb 16;29(4):881. doi: 10.3390/molecules29040881 (PMC10892774; doi:10.3390/molecules29040881)
Supplement: Supplementary file 1 [file molecules-29-00881-s001.zip › molecules-2817304-supplementary.pdf]

Supporting Information

An efficient approach to the accurate prediction of mutational effects in antigen binding to the MHC1

Mengchen Zhou[1#], Fanyu Zhao[2#], Lan Yu[3], J.F. Liu[3], Jian Wang[4] and John Z.H. Zhang[1,2,4-6*]

[1]*Shanghai Engineering Research Center of Molecular Therapeutics and New Drug Development, Shanghai Key Laboratory of Green Chemistry & Chemical Process, School of Chemistry and Molecular Engineering, East China Normal University at Shanghai, 200062, China*

[2]*NYU-ECNU Center for Computational Chemistry and Shanghai Frontiers Science Center of AI and DL, NYU Shanghai, 567 West Yangsi Road, Shanghai, 200126, China*

[3]*Department of Basic Medicine and Clinical Pharmacy, China Pharmaceutical University, Nanjing, 210009, China*

[4]*Faculty of Synthetic Biology and Institute of Synthetic Biology, Shenzhen Institute of Advanced Technology, Chinese Academy of Sciences, Shenzhen, 518055, China*

[5]*Department of Chemistry, New York University, NY, NY10003, USA*

[6]*Collaborative Innovation Center of Extreme Optics, Shanxi University, Taiyuan, Shanxi, 030006, China*

1. Effect of the number of trajectories on convergence

We used the Pearson correlation coefficient (r_P) and the Spearman ranking correlation coefficient (r_S) to rank the binding affinities. Where r_P assesses the degree of linear correlation between the two data sets, and r_S measures monotone association between the two data sets. To assess the impact of the number of trajectories on the convergence of each free energy protocol under study, we conducted a resampling with replacement (bootstrapping) using the Python software package. In 22 sets of complexes, a subset of *N* calculated values (*N* ranging from 1 to 6) was extracted from the 6 repeated MD simulations of each complex. The average of these *N* calculated values was considered as the predicted value for that specific complex. This procedure was repeated to form a set of predicted values for all 22 complexes. The process was iterated 100,000 times, and the Pearson correlation coefficient and the Spearman ranking correlation coefficient for each set of predicted values were calculated. Ultimately, this approach yielded correlation coefficients and corresponding standard deviations for different number of replicated trajectories.

Figure S3 illustrates the distribution of the correlation in relation to the number of trajectories, using the optimized ASGBIE method to obtain the calculated values (ΔE_{vdW}-$T\Delta S$). We used a bootstrap approach to sample the results of the 6 trajectories for each mutation. This approach was motivated by the fact that repeatability between trajectories is difficult to guarantee, and by sampling multiple times we can demonstrate the confidence of our results. In addition, we can get the number of trajectories needed to obtain reliable results through the distribution. As shown in Figure S3, as the number of trajectories increases, the correlation increases and the standard deviation decreases. For the Average method, the Pearson correlation coefficients and their uncertainties have reached convergence after four repetitions of the MD simulation, which is in general agreement with previous studies.[1]

The results comparing the three alanine scanning methods are consistent with the above. The Pearson coefficient improved from 0.69 ± 0.07 (MHC-AS) and 0.84 ± 0.03(peptide-AS), to 0.87 ± 0.03(Average) across the six replicas. The higher uncertainty observed in Mut-MHC method may stem from the larger standard deviations between its trajectories, as certain instances of some calculated values significantly deviate from mean. When these values are sampled for correlation calculations, the resultant correlations are notably low, with some even displaying negative values. Hence, utilizing MHC-AS approach alone might necessitate performing more simulations to obtain relatively reproducible binding free energies.

2. Reasons for the inaccuracy of the alchemical method

The inaccuracy can be attributed to several reasons. Some of the reasons originates from finite-size effect of periodic boxes.[2, 3] The exploitation of periodic boundary condition (PBC) results in the artifacts in the electrostatic potential energy calculations.[3] The periodicity of charged solute also introduces the undersolvation of

the protein-ligand complex in the reference box.[3] Furthermore, the commonly used PME[4] method introduces implicit plasma to neutralize the system and it can lead to inaccurate estimation of electrostatic potential energy of charged complex. Chen *et al.*[5]reported several approaches to correct the finite size effect of charged system, including post-simulation charge correction, solvent potential correction, co-alchemical ion, and pK_a correction.[5] Here we will briefly introduce the post-simulation charge correction adopted from Rocklin's paper. [3] It includes two correction terms to eliminate the spurious interactions between charged complex and undersolvation of the complex in the reference box. The difference between explicit water molecules used in TI simulations and the implicit solvent models used in non-periodic boundary conditions is also considered. The last correction term is residual integrated potential effect that accounts for the difference between the scenarios of a charge distribution and a naked point charge. The charge correction will be applied on the systems with net charge-changing and discussed in the future.

Figures

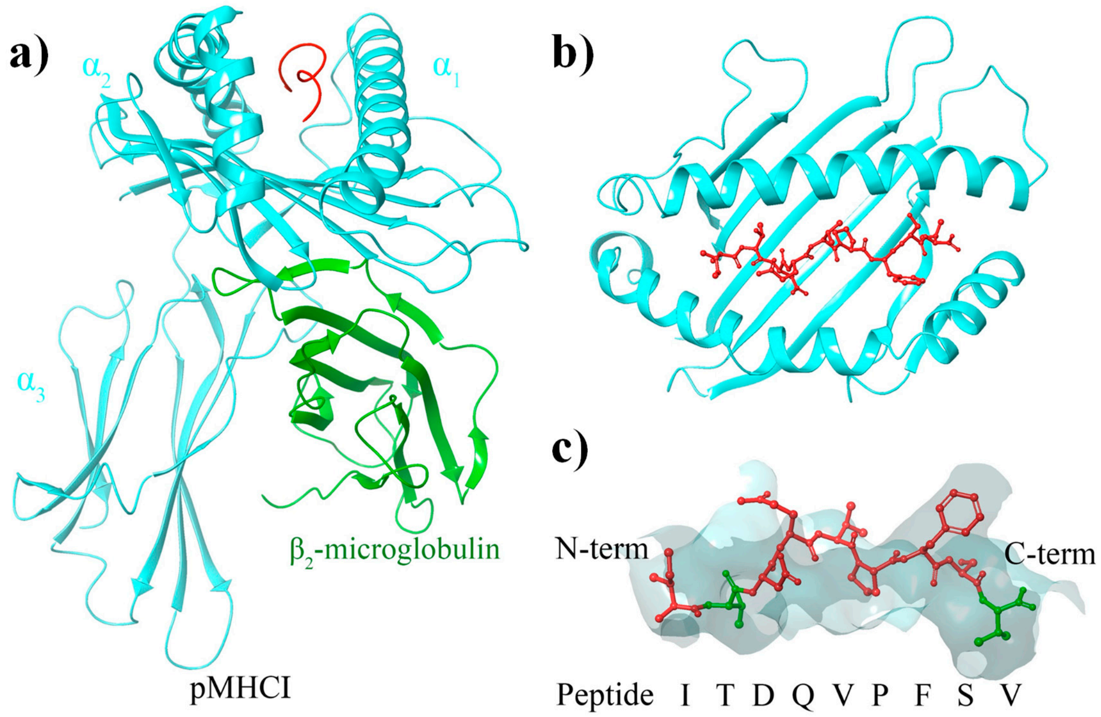

Figure S1. (a) Overview of the peptide-MHC1 complex (PDB id: 1TVB). (b) Structure of the binding interface (includes peptide and MHC1 binding groove). (c) Structure of the binding site and the anchor residue.

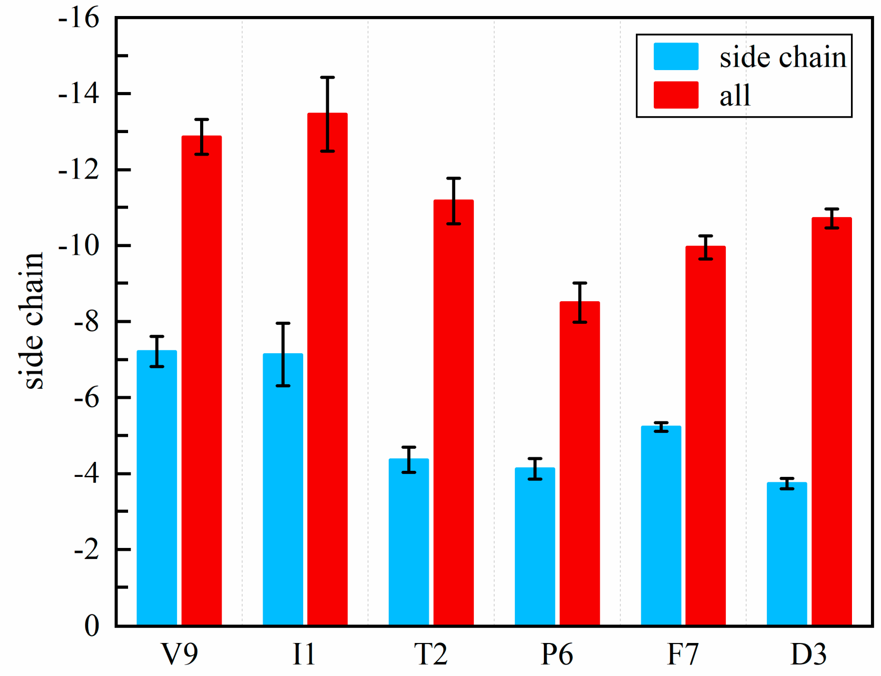

Figure S2. The vdW interactions between peptide hotspot residues and MHC groove residues: blue represents contributions from peptide side chain atoms (excluding CB and attached hydrogens), red represents contributions from all atoms in peptide residues.

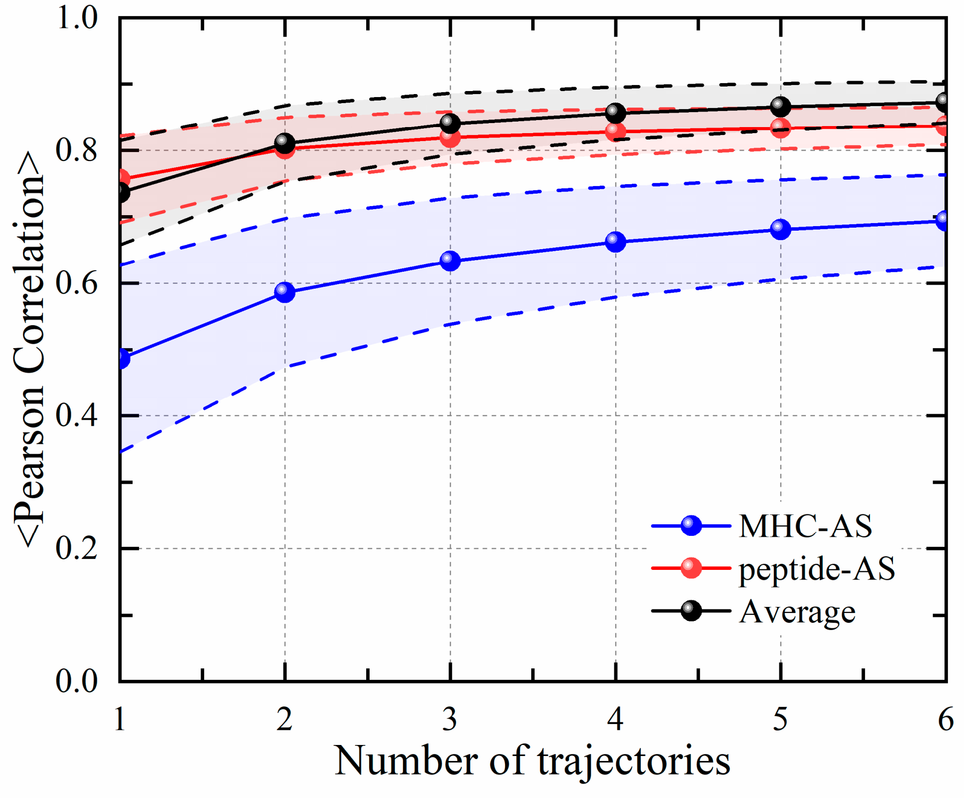

Figure S3. Bootstrapping to access the effect of the number of replicated trajectories on the Pearson correlation coefficient and its standard deviation.

Tables

Table S1. Experimental binding free energy and calculated data for MHC-AS method with wild-type and mutants.

Peptide mutation	ΔG_{exp}	$\Delta H_{MM/GBSA}$	ΔH	ΔG	ΔE_{vdW}-$T\Delta S$
wild-type	-9.28	-81.09±5.85	-67.1±3.05	-33.69±5.08	-14.72±2.22
1F	-9.9	-88.13±3.05	-68.36±1.13	-36.91±2.58	-15.42±3.46
1W	-8.43	-69.66±9.42	-59.11±5.44	-24.82±7.67	-11.52±4.63
1Y	-9.7	-79.99±4.12	-66.73±2.07	-33.24±4.35	-14.45±3.24
2I	-10.15	-76.97±4.18	-63.54±2.24	-30.4±3.52	-14.95±2.83
2L	-11.64	-80.22±4.48	-67.1±2.28	-33.75±3.55	-17.18±3.74
2M	-10.59	-77.57±7.64	-66.82±3.69	-32.54±4.77	-16±2.98
3A	-9.63	-86.12±5.14	-65.45±2.87	-34.84±3.91	-15.19±1.88
3F	-9.85	-90.88±7.94	-66.95±3.51	-35.44±4.52	-16.15±2.92
3M	-10.15	-93.31±5.52	-67.88±3.62	-32.26±6.16	-13.89±3.87
3S	-8.5	-82.17±3.92	-65.81±2.39	-34.21±2.78	-15.02±1.95
3W	-10.24	-91.69±2.52	-65.35±1.56	-31.47±2.62	-13.35±2.73
3Y	-10.27	-91±5.86	-66.31±4.01	-33.64±4.4	-14.71±2.84
1F2L	-11.89	-83.82±3.85	-68.09±2.59	-40.79±2.04	-21.92±1.41
1W2L	-10.9	-76.11±12.19	-60.98±10.24	-29.63±9.94	-15.29±5.95
1Y2L	-11.86	-82.65±1.89	-69.31±1.43	-40.06±2.18	-22.69±1.97
2L3A	-10.9	-85.41±7.13	-65.55±2.18	-34.38±2.59	-17.14±2.38
2L3F	-11.94	-84.29±3.48	-65.16±2.25	-35.32±3.92	-19.36±3.38
2L3M	-11.15	-89.68±5.09	-67.52±3.21	-34.6±4.83	-17.64±3.56
2L3S	-10.57	-81.89±4.7	-65.51±2.95	-33.99±4.13	-17.07±2.58
2L3W	-12.04	-87.86±3.29	-65.02±3.1	-32.58±3.84	-16.56±3.45
2L3Y	-11.4	-85.69±4.55	-65.18±2.1	-32.74±4.96	-17.87±2.82
Pearson Correlation		0.22	0.30	0.46	0.77
Spearman Correlation		0.12	0.05	0.22	0.78

[a]All energies in kcal/mol

Table S2. Experimental binding free energy and calculated data for peptide-AS method with wild-type and mutants.

Peptide mutation	ΔG_{exp}	$\Delta H_{MM/GBSA}$	ΔH	ΔG	ΔE_{vdW}-$T\Delta S$
wild-type	-9.28	-81.09±5.85	-32.1±0.94	-23.16±1.51	-21.92±1.47
1F	-9.9	-88.13±3.05	-32.83±0.54	-24.19±1.35	-22.59±1.06
1W	-8.43	-69.66±9.42	-35.48±3.13	-24.46±3.41	-24.54±2.88
1Y	-9.7	-79.99±4.12	-32.56±1.04	-23.51±2.48	-22.93±2.05
2I	-10.15	-76.97±4.18	-33.9±1.38	-24.89±2.64	-26.25±2.49
2L	-11.64	-80.22±4.48	-34.24±0.46	-25.45±1.88	-27.97±2.16
2M	-10.59	-77.57±7.64	-33.64±1.28	-22.02±3.83	-25.1±3.84
3A	-9.63	-86.12±5.14	-29.85±1.4	-20.72±2.29	-19.02±2.07
3F	-9.85	-90.88±7.94	-35.83±1.11	-26.88±1.56	-26.43±1.11
3M	-10.15	-93.31±5.52	-36.68±0.49	-25.08±0.9	-24.58±0.82
3S	-8.5	-82.17±3.92	-31.06±1.99	-21.04±2.69	-20.32±2.79
3W	-10.24	-91.69±2.52	-36.38±1.77	-25.77±2.2	-25.87±2.58
3Y	-10.27	-91±5.86	-37.96±0.88	-26.69±3.15	-25.02±2.49
1F2L	-11.89	-83.82±3.85	-35.28±0.6	-28.09±1.35	-30.34±0.87
1W2L	-10.9	-76.11±12.19	-38.78±1.7	-27.42±3.15	-28.51±3.17
1Y2L	-11.86	-82.65±1.89	-36.31±0.34	-28.99±0.69	-31.46±0.97
2L3A	-10.9	-85.41±7.13	-33.26±1.04	-24.15±1.75	-25.75±1.57
2L3F	-11.94	-84.29±3.48	-38.52±1.07	-28.58±2.02	-32.11±1.93
2L3M	-11.15	-89.68±5.09	-37.61±1.7	-26.35±1.92	-29.25±1.45
2L3S	-10.57	-81.89±4.7	-33.57±1.32	-23.89±2.53	-25.58±2.59
2L3W	-12.04	-87.86±3.29	-38.97±1.15	-29.61±1.71	-33.93±1.87
2L3Y	-11.4	-85.69±4.55	-40.21±1.05	-31.5±2.13	-33.18±1.83
Pearson Correlation		0.22	0.58	0.72	0.86
Spearman Correlation		0.12	0.62	0.73	0.88

[a]All energies in kcal/mol

Table S3. Experimental binding free energy and calculated data for Average method with wild-type and mutants.

Peptide mutation	ΔG_{exp}	$\Delta H_{MM/GBSA}$	ΔH	ΔG	ΔE_{vdW}-$T\Delta S$
wild-type	-9.28	-81.09±5.85	-49.6±1.91	-28.42±3.24	-18.32±1.59
1F	-9.9	-88.13±3.05	-50.59±0.75	-30.55±1.43	-19±1.9
1W	-8.43	-69.66±9.42	-47.3±3.02	-24.64±4.48	-18.03±3.11
1Y	-9.7	-79.99±4.12	-49.65±1.47	-28.38±3.17	-18.69±2.38
2I	-10.15	-76.97±4.18	-48.72±1.68	-27.65±2.04	-20.6±1.49
2L	-11.64	-80.22±4.48	-50.67±1.21	-29.6±2.09	-22.58±2.69
2M	-10.59	-77.57±7.64	-50.23±2.25	-27.28±3.97	-20.55±3.24
3A	-9.63	-86.12±5.14	-47.65±1.9	-27.78±2.55	-17.11±1.62
3F	-9.85	-90.88±7.94	-51.39±2.26	-31.16±2.69	-21.29±1.49
3M	-10.15	-93.31±5.52	-52.28±1.79	-28.67±2.99	-19.24±1.81
3S	-8.5	-82.17±3.92	-48.44±1.97	-27.62±2.39	-17.67±1.69
3W	-10.24	-91.69±2.52	-50.86±1.51	-28.62±2.11	-19.61±2.45
3Y	-10.27	-91±5.86	-52.14±2.39	-30.17±3.03	-19.86±2.03
1F2L	-11.89	-83.82±3.85	-51.69±1.41	-34.44±1.52	-26.13±0.9
1W2L	-10.9	-76.11±12.19	-49.88±5.86	-28.53±6.19	-21.9±4.1
1Y2L	-11.86	-82.65±1.89	-52.81±0.73	-34.52±0.96	-27.07±1.29
2L3A	-10.9	-85.41±7.13	-49.4±1.27	-29.27±1.86	-21.45±1.51
2L3F	-11.94	-84.29±3.48	-51.84±1.33	-31.95±2.33	-25.74±2.32
2L3M	-11.15	-89.68±5.09	-52.57±2	-30.47±2.58	-23.45±1.57
2L3S	-10.57	-81.89±4.7	-49.54±2.11	-28.94±3.19	-21.33±2.51
2L3W	-12.04	-87.86±3.29	-51.99±1.95	-31.1±2.01	-25.25±1.81
2L3Y	-11.4	-85.69±4.55	-52.69±1.23	-32.12±2.93	-25.53±1.95
Pearson Correlation		0.22	0.71	0.75	0.91
Spearman Correlation		0.12	0.65	0.71	0.94

[a]All energies in kcal/mol

Table S4. The vdW interaction of peptide side chain with MHC groove.

Residue on peptide	Residue on MHC groove	VdW interaction energy (kcal/mol)
V9	77D	-1.46
	116Y	-1.16
	123Y	-0.74
	81L	-0.73
	80T	-0.61
I1	167W	-1.82
	163T	-0.83
	159Y	-0.58
	63E	-0.55
T2	7Y	-0.89
	66K	-0.64
	70H	-0.54
P6	70H	-1.25
	97R	-1.04
	73T	-0.66
F7	152V	-1.42
	147W	-0.95
	150A	-0.83
	146K	-0.59
D3	159Y	-1.74
	156L	-0.58

* The side chains of the peptides did not include the CB and the attached hydrogens, and only residues with vdW interaction energies less than -0.5 kcal/mol were listed.

Table S5. Spearman correlation coefficient for each energy with experimental binding affinity under different alanine scanning methods. MHC-AS means the ASGBIE calculation is calculated based on the summation over MHC residues, and similar definition holds for Peptide-AS method.

**Energy component**	**Spearman Correlation Coefficient**	
	**MHC-AS**	**Peptide-AS**
$\Delta H_{MM/GBSA}$	0.12	0.12
ΔE_{vdW}	0.71	0.84
ΔH	0.05	0.62
ΔG	0.22	0.73
$\Delta E_{vdW} - T\Delta S$	0.78	0.88

Table S6. The experimental and calculated data of relative binding free energy of 9 mutations without net charge-changing.

Peptide modification	$\Delta\Delta G_{exp}$	Alchemical method	Optimized ASGBIE
1F	-0.61	-0.82±0.2	-0.69±2.2
1W	0.85	0.36±0.12	0.29±3.92
1Y	-0.41	-0.36±0.12	-0.37±2.64
2I	-0.87	-1.04±0.21	-2.28±1.34
2L	-2.35	-2.34±0.08	-4.26±3.49
2M	-1.31	-1.98±0.11	-2.23±3.02
1F2L	-2.6	-2.25±0.14	-7.81±1.28
1W2L	-1.61	-1.72±0.49	-3.58±4.46
1Y2L	-2.58	-2.81±0.3	-8.75±2.26
Pearson Correlation		0.97	0.90
Spearman Correlation		0.93	0.97

Table S7. The experimental and calculated data of relative binding free energy of 12 mutations with net charge-changing.

Peptide modification	$\Delta\Delta G_{exp}$	Alchemical method	Optimized ASGBIE
3A	-0.35	-1.92±0.91	1.21±2.84
3F	-0.57	-4.48±1.02	-2.97±1.75
3M	-0.87	-8.54±0.42	-0.92±1.87
3S	0.78	-2.79±1.93	0.65±2.48
3W	-0.96	-2.81±0.49	-1.29±2.27
3Y	-0.98	-5.55±0.07	-1.54±2.19
2L3A	-1.61	1.61±2.09	-3.13±2.39
2L3F	-2.65	-4.87±2.73	-7.42±2.67
2L3M	-1.87	-1.92±0.67	-5.13±1.81
2L3S	-1.28	-0.62±0.82	-3.01±2.76
2L3W	-2.76	-3.79±0.87	-6.93±2.91
2L3Y	-2.12	-3.89±1.93	-7.21±2.53
Pearson Correlation		-0.01	0.91
Spearman Correlation		0.02	0.93

References

1. Knapp, B.; Ospina, L.; Deane, C. M., Avoiding False Positive Conclusions in Molecular Simulation: The Importance of Replicas. *Journal of chemical theory and computation* **2018**, 14, 6127-6138.
2. Hünenberger, P. H.; McCammon, J. A., Ewald artifacts in computer simulations of ionic solvation and ion–ion interaction: A continuum electrostatics study. *The Journal of Chemical Physics* **1999**, 110, 1856-1872.
3. Rocklin, G. J.; Mobley, D. L.; Dill, K. A.; Hünenberger, P. H., Calculating the binding free energies of charged species based on explicit-solvent simulations employing lattice-sum methods: an accurate correction scheme for electrostatic finite-size effects. *J Chem Phys* **2013**, 139, 184103.
4. Essmann, U.; Perera, L.; Berkowitz, M. L.; Darden, T.; Lee, H.; Pedersen, L. G., A smooth particle mesh Ewald method. *The Journal of Chemical Physics* **1995**, 103, 8577-8593.
5. Chen, W.; Deng, Y.; Russell, E.; Wu, Y.; Abel, R.; Wang, L., Accurate Calculation of Relative Binding Free Energies between Ligands with Different Net Charges. *Journal of chemical theory and computation* **2018**, 14, 6346-6358.
